# The intrinsic geometry of the human brain connectome

**DOI:** 10.1007/s40708-015-0022-2

**Published:** 2015-11-07

**Authors:** Allen Q. Ye, Olusola A. Ajilore, Giorgio Conte, Johnson GadElkarim, Galen Thomas-Ramos, Liang Zhan, Shaolin Yang, Anand Kumar, Richard L. Magin, Angus G. Forbes, Alex D. Leow

**Affiliations:** 1 Department of Bioengineering, University of Illinois at Chicago, 218 SEO, 851 S Morgan St, Chicago, 60607 IL USA; 2 Department of Psychiatry, University of Illinois at Chicago, Chicago, IL USA; 3 Department of Computer Science, University of Illinois at Chicago, Chicago, IL USA; 4 Computer Engineering Program, Engineering and Technology Department, University of Wisconsin-Stout, Menomonie, WI USA; 5 Department of Radiology, University of Illinois at Chicago, Chicago, IL USA

**Keywords:** Diffusion MRI, Tractography, Dimensionality reduction, Virtual reality, Connectomics

## Abstract

This paper describes novel methods for constructing the intrinsic geometry of the human brain connectome using dimensionality-reduction techniques. We posit that the high-dimensional, complex geometry that represents this intrinsic topology can be mathematically embedded into lower dimensions using coupling patterns encoded in the corresponding brain connectivity graphs. We tested both linear and nonlinear dimensionality-reduction techniques using the diffusion-weighted structural connectome data acquired from a sample of healthy subjects. Results supported the nonlinearity of brain connectivity data, as linear reduction techniques such as the multidimensional scaling yielded inferior lower-dimensional embeddings. To further validate our results, we demonstrated that for tractography-derived structural connectome more influential regions such as rich-club members of the brain are more centrally mapped or embedded. Further, abnormal brain connectivity can be visually understood by inspecting the altered geometry of these three-dimensional (3D) embeddings that represent the topology of the human brain, as illustrated using simulated lesion studies of both targeted and random removal. Last, in order to visualize brain’s intrinsic topology we have developed software that is compatible with virtual reality technologies, thus allowing researchers to collaboratively and interactively explore and manipulate brain connectome data.

## Introduction

Magnetic resonance imaging (MRI) techniques have allowed us to noninvasively study the human brain both anatomically and functionally. The complex interactions between different regions of the brain have necessitated the development and growth of the field of connectomics. A brain connectome at the macroscale is typically mathematically represented with connectivity matrices that describe the interaction between the different brain regions. Most current connectome study designs are based on brain connectivity matrices, which involve the computation of summary statistics on a global or nodal level [1]. However, current connectome visualization methods typically represent anatomic and functional connectivity data using somewhat arbitrary or heuristic methods [2]. In this study, we address this shortcoming by developing a framework that realizes, constructs, and visually represents the complex intrinsic geometry or topology of the entire brain network.

Classical linear techniques for dimensionality reduction such as principal component analysis (PCA) and multidimensional scaling (MDS) are computationally efficient and suitable for linear structures [3]. However, neural networks represent highly nonlinear data and may exhibit more complexity than what PCA or MDS are designed to detect [4].

Thus, we propose to use nonlinear dimensionality-reduction algorithms that maintain the advantages of PCA or MDS, namely, computational efficiency, global optimality, and asymptotic convergence guarantees. Nonlinear dimensionality reduction solves the well-known “Swiss roll problem,” where the shortest Euclidean distances between data points at a higher-embedded dimension are not representative of the actual geodesic path along the low-dimensional manifold i.e., the intrinsic geometry (in the case of the Swiss roll, the intrinsic geometry is a 2D plane which is rolled up in a 3D space). Intuitively, this was accomplished in Isomap [5] by modifying the classical MDS. Isomap reconstructs the path length for points that are far away by adding up a series of steps between nodes to approximate its geodesic distance. As a result, Isomap was described by the authors to be a “complete isometric feature mapping.”

In recent years, other dimensionality-reduction techniques have been proposed and examined for comparisons versus Isomap (e.g., locally linear embedding (LLE) [6], Laplacian eigenmaps [7], diffusion maps [8], and t-distributed stochastic neighbor embedding (t-SNE) [9]). One goal of this paper is to examine the intrinsic geometry of the brain and to see if “crowding” of data points in the lower-dimensional embedding is an issue that requires more advanced dimensionality-reduction techniques.

Clinically, previous work with nonlinear dimensionality reduction has been applied to large datasets consisting of a combination of imaging and non-imaging data (lab measurements, gene sequencing), assembled to develop accurate biomarkers to better understand disease progression [10]. t-SNE in particular has been used to explore areas such as breast cancer [11] and proteomics [12].

To the best of our knowledge, this paper represents the first application of dimensionality reduction to reveal the brain connectivity’s intrinsic geometry. To put into context why the intrinsic geometry may be a better space to understand brain connectivity data, we can look at the field of cartography. For decades, cartographers have mapped quantitative data onto world maps to create unique, informative visualizations. For example, by resizing regional areas of the state of New York according to the incidence of lung cancer, one can show graphically that New York City occupies the largest area and therefore has the highest incidence of lung cancer [13]. Similarly, dimensionality-reduction techniques remap the brain according to its connectivity such that in the resulting geometry the shape the connectome assumes is independent of the anatomic distances between nodes. Using tractography-derived structural connectomes to illustrate this point, the proposed approach relies on the intuition that, as long-range fiber pathways (e.g., the superior longitudinal fasciculus or SLF) connect brain regions that are physically relatively far apart, its topology may thus be better determined using the corresponding connectivity matrix, rather than the inter-regional anatomic distances.

## Methods

### Image acquisition

Forty-six healthy control subjects (HC, mean age: 59.7 ± 14.6, 20 males) were recruited by community outreach using newspaper, radio, television advertisements, and relevant outpatient clinics. The study was approved by the University of Illinois at Chicago Institutional Review Board and conducted in accordance with the Declaration of Helsinki.

We acquired brain MRI data on a Philips 3.0T Achieva scanner (Philips Medical Systems, Best, The Netherlands) using an 8-channel SENSE (sensitivity encoding) head coil. High-resolution three-dimensional (3D) T1-weighted images were acquired with a MPRAGE (magnetization prepared rapid acquisition gradient echo) sequence (field of view: FOV = 240 mm; 134 contiguous axial slices; TR/TE = 8.4/3.9 ms; flip angle = 8°; voxel size = 1.1 × 1.1 × 1.1 mm). For DTI images, we used single-shot spin-echo echo-planar imaging (EPI) sequence (FOV = 240 mm; voxel size = 2.5 × 2.5 × 2.2 mm that was interpolated to 0.83 × 0.83 × 2.2 mm; TR/TE = 6,994/71 ms; flip angle = 90^o^). Sixty seven contiguous axial slices aligned to the anterior commissure–posterior commissure (AC-PC) line were collected in 32 gradient directions with *b* = 700 s/mm^2^ and one acquisition without diffusion sensitization (B0 image). Parallel imaging was utilized with an acceleration factor of 2.5 to reduce scanning time to approximately 4 min.

### Data preprocessing

We generated individual structural brain networks for each of the forty-six subjects using a pipeline reported previously [14]. First, diffusion-weighted (DW) images were eddy current corrected using the automatic image registration (AIR) tool embedded in DtiStudio software (http://www.mristudio.org), by registering all DW images to their corresponding B_0_ images with 12-parameter affine transformations. This was followed by computation of diffusion tensors and deterministic tractography using fiber assignment by a continuous tracking algorithm [15]. T1-weighted images were used to generate label maps using the Freesurfer software (http://surfer.nmr.mgh.harvard.edu).

A total of 82 Freesurfer labels were created for structural images and then further subdivided using an algorithm that continuously bisected each region across all subjects using a plane perpendicular to the main axis of its shape. Mathematically, this is achieved by first aligning the centroid coordinates of this ROI across all subjects to yield a combined group ROI (thus accounting for the difference in individual subject spaces). Second, we determined the main axis by conducting a PCA on all voxels belonging to this combined group ROI. Previous studies using similar algorithms have shown that upsampling regions into higher-resolution voxels maintains network connectivity [16]. In this study, we chose a threshold of 800 voxels or about 1 cm^3^; when the size of an ROI dropped below this threshold, it would no longer be further subdivided. This procedure upsampled the overall gray matter regions by about 8 times, thus converting the structural 82 regions into 620 sub regions, resulting in structural brain network connectivity matrices of size 620 by 620. All networks were examined to ensure that all regions were directly connected to at least one other region preventing the formation of any isolated “islands.” To compensate for inter-subject variations, we averaged all individuals’ networks together to obtain a group average network.

### Dimensionality reduction

In this study, we mainly discussed results generated using two classic dimensionality-reduction techniques: (1) MDS—a classic linear embedding technique, (2) Isomap, a nonlinear dimensionality reduction as described by [5]. A modern nonlinear embedding technique called t-distributed stochastic neighborhood embedding [9] was additionally tested as it has been theorized to be advantageous over Isomap, but was found to be inferior to Isomap in our specific application and thus was excluded from this paper.

After the structural networks are generated, the corresponding connectome data need to be appropriately represented in a high-dimensional space where a distance metric can be properly computed (such that a neighborhood can be defined for constructing the Isomap).

Here, we propose to represent connectivity data in a *n* = 620 Euclidean space by placing node *i* at the coordinates defined by the vector *d* that codes its graph distance to every node in the brain: $d_{i} = ({\text{GraphDistance}}_{i,1} , {\text{GraphDistance}}_{i,2} \ldots , {\text{GraphDistance}}_{i,n} )$; here GraphDistance represents the shortest path length between two nodes; the graph distance matrix of a structural connectome is usually formed by defining edge length as the inverse of the connectivity strength followed by applying the Dijkstra’s algorithm. To understand why this would realize the intrinsic geometry of the structure connectome, we simply note that in the intrinsic geometry one would want to embed two nodes (*k* and *l*) next to each other if $\parallel d_{k} - d_{l} \parallel = \sqrt {\mathop \sum \limits_{i} ({\text{GraphDistance}}_{k,i} - {\text{GraphDistance}}_{l,i} ) ^{2} }$ is small.

To promote uniformity throughout the analyses, we used the dimensionality-reduction toolbox introduced by van der Maaten for all reductions [17]. The number of dimensions was reduced from 620 to 3 dimensions. For Isomap, the number of nearest numbers used for neighborhood determination was increased iteratively until all regions were included during manifold building. For both structural and functional imaging modes, this step created the final output.

### Visualization using BRAINtrinsic

**Fig. 1:**
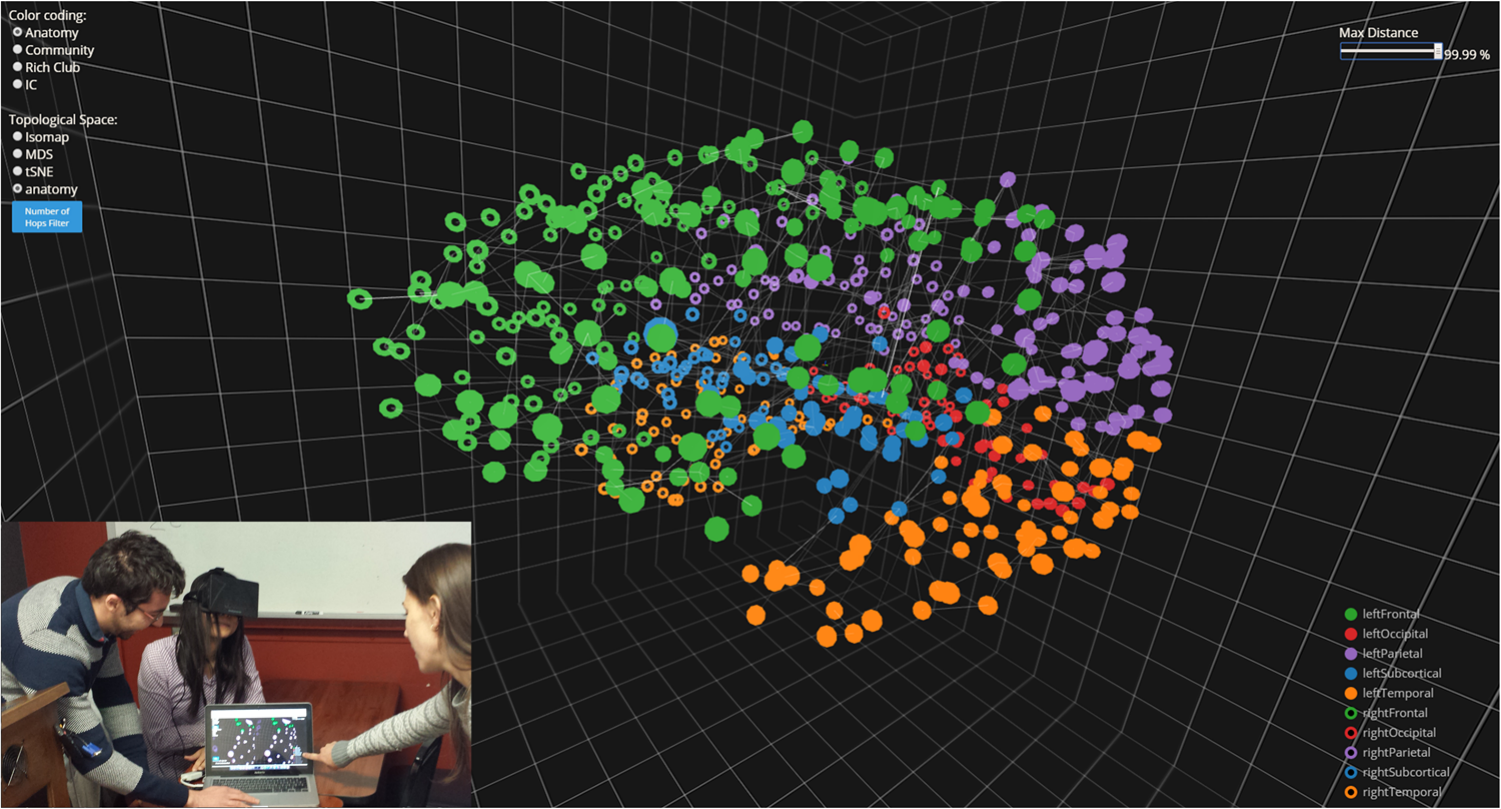
The anatomy of a human brain shown in BRAINtrinsic. Different *colors* represent different lobes of the brain, while the line segments show shortest paths connecting brain nodes (e.g., in tractography-derived structural connectome, the thicker the line, the more reconstructed tracts can be seen in the connectivity matrix). *Inset* shows the author (Leow) using the Oculus Rift technology to visualize brain connectome data using the proposed approach

In order to visualize and navigate a 3D embedding of the intrinsic geometry, we must have a flexible and robust viewing platform. To this end, we have developed BRAINtrinsic, an open source virtual reality visualization system. BRAINtrinsic exploits the hardware-accelerated graphics functionality provided by WebGL (www.webgl.com) and is designed to be fully compatible within a virtual reality environment. Presently, BRAINtrinsic can be also used with the Oculus Rift device (www.oculus.com). The code is open source and publicly available at the authors’ code repository at (https://github.com/gioconte/gioconte.github.io). An example of the layout and graphical user interface can be seen in Fig. 1.

### Targeted node removal

Previous studies have looked at the differences in brain network robustness and its tolerance to removal of nodes (either targeted or random) [18], including the removal of rich-club regions [19, 20]. To test if dimensionality-reduction techniques capture visually meaningful and interpretable information in a 3D environment, we used similar removal strategies to understand the structural connectome’s topology after random or targeted node removal.

Our metric of choice is $\bar{d}$, the average Euclidean distance from all embedded nodes to the center of the embedding. As a primary investigation we removed rich-club regions as previously defined in [21], which consist of the left and right precuneus, superior frontal cortex, superior parietal cortex, hippocampus, putamen, and thalamus. These twelve regions represented 21.5 % of all nodes in our 620-region structural brain connectome. For comparison, we then ran 20,000 trials that randomly selected and removed the same amount of nodes to obtain a distribution of $\bar{d}$ under random removal.

Finally, we also removed the first 21.5 % of nodes using various targeted removal schemes according to the following well established connectome measures: (a) nodal strength (descending), (b) clustering (ascending), (c) nodal path length (ascending), and (d) betweenness centrality (descending) [22], as well as e) embeddedness, a recently proposed novel connectome metric [23]. Embeddedness computes a ratio between nodal efficiency and the rate of information transfer decay for this node, and thus probes the relative scale-invariant information exchange efficiency. Highly embedded brain regions are those that comprise the limbic system, the default mode network, and the subcortical nuclei. These regions are linked in the evolutionary role they play in memory, emotion and behavior.

## Results

### High-dimensional representation of functional and structural brain connectivity

**Fig. 2:**
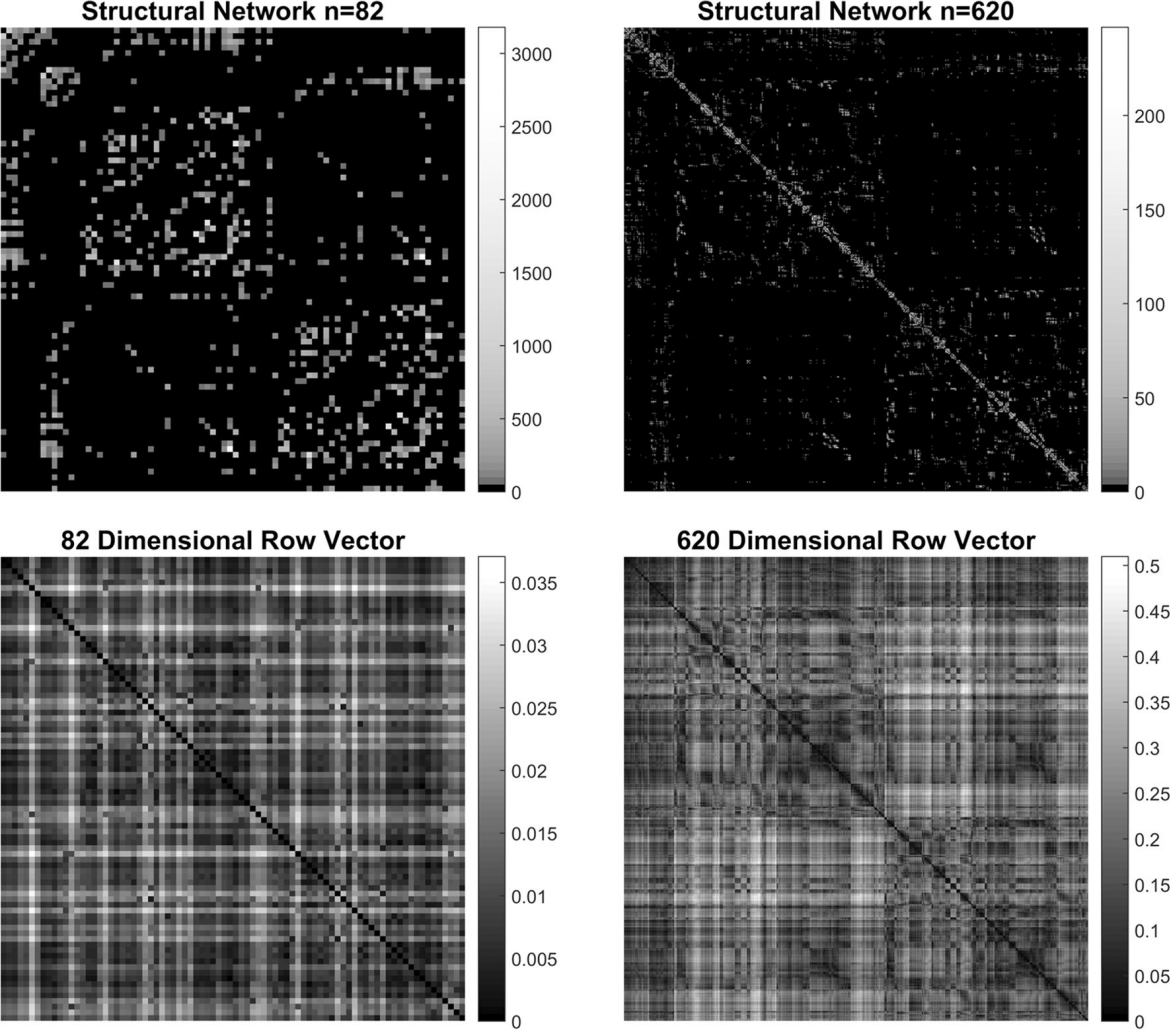
Connectivity matrices for the original structural (*n* = 82 nodes) and upsampled structural (*n* = 620 nodes) connectomes. For the structural networks, the (*i*, *j*) element represents the tractography-based fiber count between brain regions, *i* and *j*. The resulting n-dimensional *row vectors* (*vertically stacked*; *bottom row*) describes the high-dimensional Euclidean representations of connectome data

Figure 2 shows the adjacency matrices for both the structural group-averaged connectome (Top row). The (*i, j*) element represents the tractography-based fiber count between brain regions *i* and *j*. The resulting row vectors (vertically stacked; bottom row) computed using the procedure in 2.3 now describes the proposed high-dimensional Euclidean representation of the brain connectome. Here, each row represents the Euclidean coordinates of the corresponding brain region (620 dimensions for structural data). Note that in the structural data, these vertically stacked row vectors are equivalent to the graph distance matrix of the structural adjacency matrix.

### Creation of the MDS brain

**Fig. 3:**
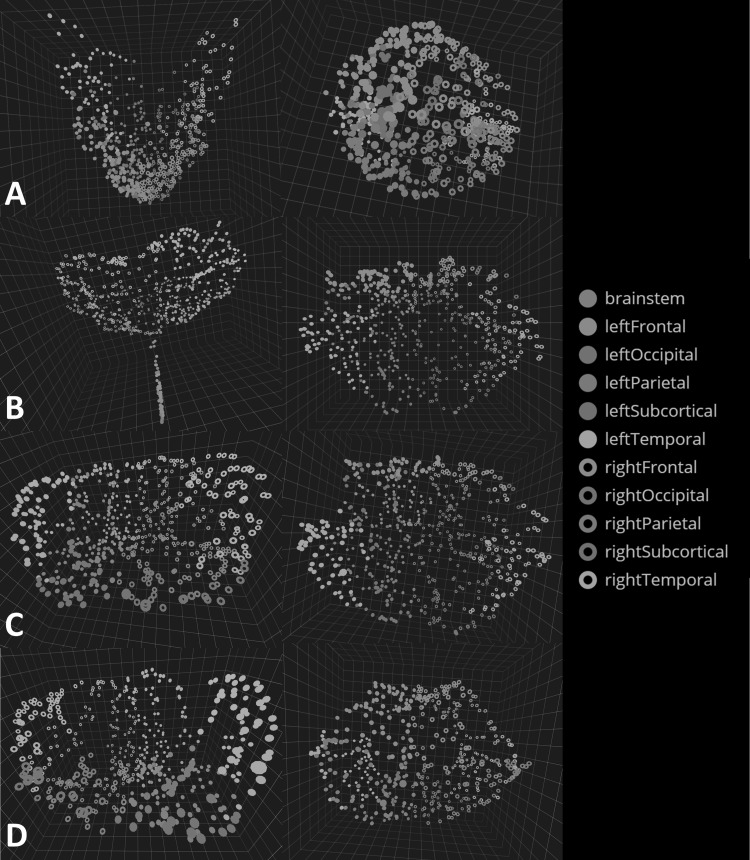
**a** BRAINtrinsic visualization of the transformed MDS brain map with a coronal view (*left*) and a top or axial view (*right*). *Colors* represent different lobes of the brain. Visually, MDS embedding resembles a funnel with a high concentration of nodes near the vertex. **b** The Isomap topology of the structural brain connectome formed by cortical/subcortical gray matter regions plus the brain stem. Note here brainstem and several subcortical regions are closely embedded in the Isomap, suggesting that they are highly interconnected (while less so with the rest of the brain), which is consistent with known neuroanatomy. **c** The Isomap topology for the connectome formed by the cortical/subcortical gray matter regions without the brain stem from a coronal (*left*) and axial (*right*) viewpoint. Visually resembling a flower, this topology and its simple Euclidean quantifications in Figs. 5 and 6 provide intuitive insight into the relative role each brain region plays, as well as how removal of targeted regions can affect this configuration. We additionally examined the connectome’s topology after removing subcortical structures (**d**), with results suggesting that in the absence of subcortical regions, the topology remains minimally altered, likely thanks to alternative routing via cortico-cortical connections

MDS, a classic linear technique, was first used to test the proposed visualization platform. Visually, the MDS embedding of the structural connectome primarily clustered into a funnel shape (Fig. 3a). As will be shown in the following section, there are clear advantages in the realized embedding as we move from MDS, a linear dimensionality technique, to Isomap, a nonlinear dimensionality-reduction technique.

### Creation of the Isomap brain

Figure 3b–d shows the intrinsic topology of the structural connectome in the Isomap space. Three variations were visualized: the entire brain connectome formed by the 82 cortical/subcortical gray matter brain regions plus the brain stem (Fig. 3b), the brain connectome formed by the 82 cortical/subcortical gray matter regions (Fig. 3c), and the brain connectome formed by 68 cortical regions alone (Fig. 3d). Here, the shape of the computed brain Isomaps visually resembles a parabolic bowl or a flower, with the brain stem as the stem and the other lobes forming the inner and outer petals.

Visually, the intrinsic geometry of the structural connectome formed by 68 cortical gray matter ROIs alone does not differ much from that of the connectome formed by both cortical and subcortical gray matter ROIs. Contrasting this with the targeted removal results shown in later sections, one may argue that in the absence of subcortical structures interhemispheric communication remains minimally impacted, likely via cortico-cortical connections [24].

### Fundamental network measures for Embeddings

One way to validate the utility of dimensionality reduction, especially in the case of structural connectome, is to understand the concept of nodal path length in the resulting 3D embedding. To this end, as nodes with shorter nodal path lengths communicate more efficiently with the rest of the brain and are thus more “important,” we expect that they would thus be more centrally located in the dimensionally reduced embeddings. Therefore, we hypothesize the existence of a positive correlation between the nodal path length of a node and its Euclidean distance to the origin of embedding.

**Fig. 4:**
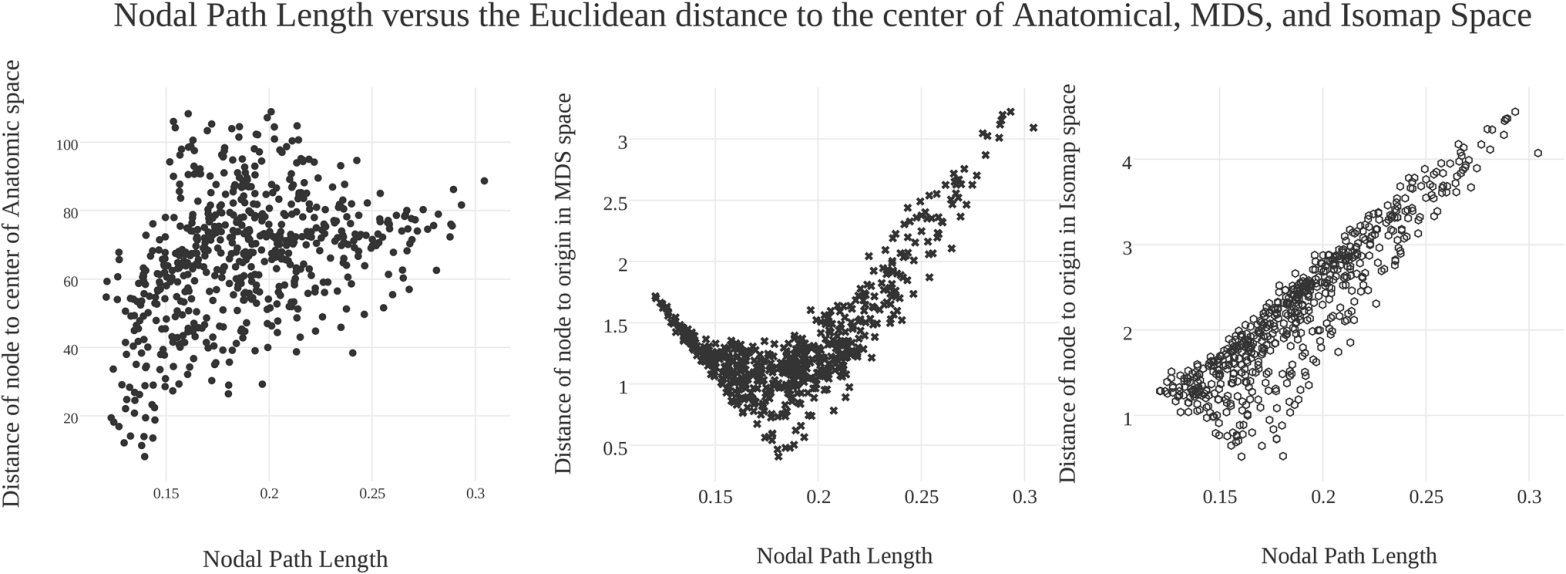
The comparison of the nodal path length (*x* axis) for the 620-ROI structural connectome versus their distances to the center in various spaces. The correlation coefficients for each subplot is *r* ^2^ = 0.144 (anatomic space), *r* ^2^ = 0.427 (MDS), and *r* ^2^ = 0.828 (Isomap). As expected, a node’s nodal path length does not relate to its distance to the brain’s anatomic center (*left subplot*), but overall, mostly linearly maps onto its Euclidean distance to the embedding’s topological center when a nonlinear technique such as Isomap is used. Indeed, as the dimensionality-reduction technique becomes more advanced (from no dimensionality reduction to linear reduction to nonlinear reduction), better representations of the intrinsic geometry are achieved and embedded in a 3D Euclidean space

Figure 4 demonstrates such a correlation (or lack of) when comparing nodal path length versus the anatomic, MDS, and Isomap ROI distance to the center of that particular embedding. The center of the anatomic brain was defined as the mean of the (*x*, *y*, *z*) coordinates of all regions-of-interest. As expected, the correlations between the nodal path length and this node’s Euclidean distance to the embedding’s origin for both dimensionality-reduction techniques (*r*^2^ of 0.427 and 0.828 for MDS and Isomap, respectively) were markedly higher than the correlation between nodal path length and this ROI’s anatomic distance to the center of the brain (*r*^2^ = 0.144). Due to the superiority of Isomap compared to MDS, we will only present results from the Isomap algorithm from here forward.

### Rich-club connectivity

**Fig. 5:**
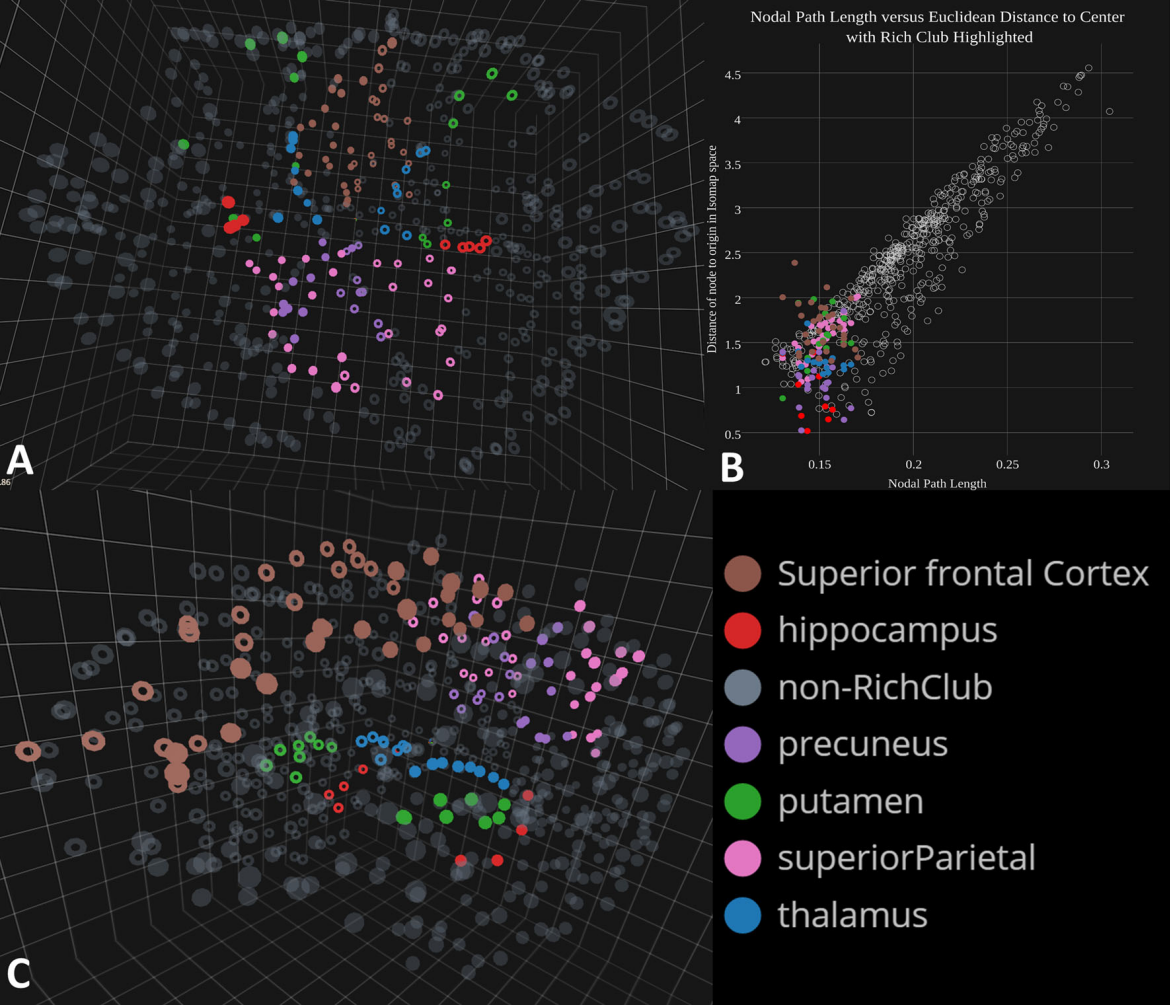
Illustration of relative locations of rich-club brain regions. **a** The Isomap embedding shown axially with the rich-club brain regions highlighted. Nodes from all six rich-club regions are centrally located with respect to the whole brain in the Isomap and are clustered close to one another. This is emphasized in **b** showing the scatter plot recreated from Fig. 4 (*right panel*), this time highlighting the rich-club nodes. **c** Neuroanatomic locations of the same rich-club regions are compared. For all panels, different colors represent different rich-club regions of the brain, while gray nodes represent nonrich-club members

**Fig. 6:**
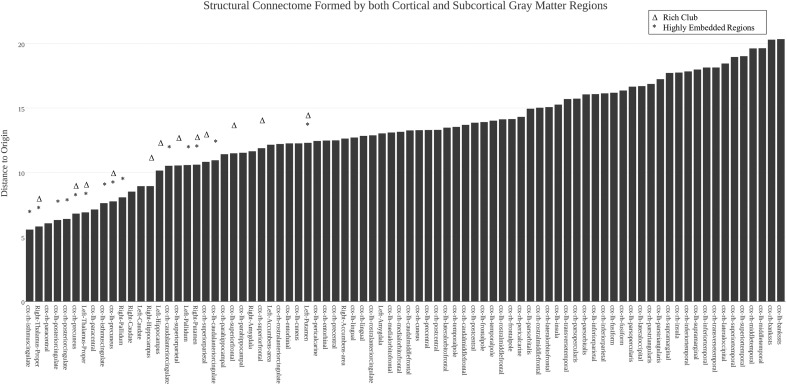
For the structural connectome formed by all 82 cortical/subcortical gray matter ROIs, the distance to the origin of the Isomap space was computed and ranked from low (*left*) to high (*right*), with each region’s name shown on the *x* axis. Rich-club and highly embedded regions are highlighted by *asterisk* and* delta*, respectively

Recent studies have suggested that certain brain regions form a “rich-club” subnetwork, such that members in this subnetwork are densely interconnected with each other, even more than expected from nodes of their degree [21]. The rich-club concept was adopted from applications in social science and computer science where certain highly central individuals or nodes were found to exist in tightly interconnected communities [25]. Brain regions that exhibit this “rich-club” property include the bilateral precuneus, superior frontal cortex, superior parietal cortex, hippocampus, putamen, and thalamus. Here, we investigate the locations of these rich-club regions in the corresponding brain Isomap. Results suggest that, indeed, nodes from all six rich-club regions are visually centrally located in the Isomap (Fig. 5); moreover, they are also clustered close to one another. On the other hand, as shown in Fig. 6, we note that interestingly not all centrally located regions are traditionally labeled rich club (e.g., the left and right caudate, pallidum, paracentral lobule, and caudal anterior, posterior, and isthmus cingulate are all relatively centrally located).

**Fig. 7:**
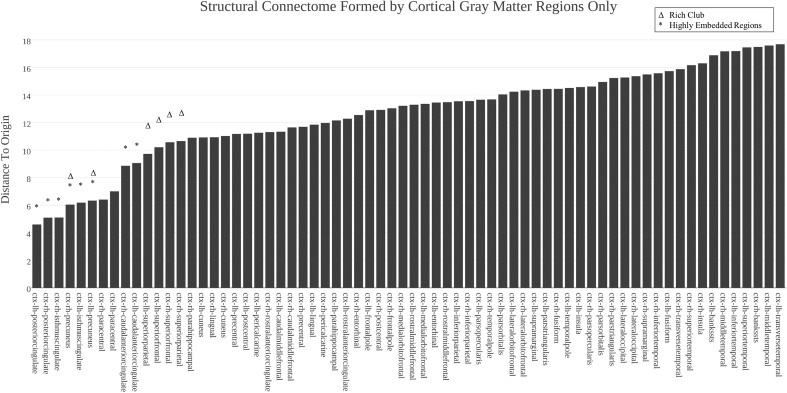
For the structural connectome formed by 68 cortical gray matter ROIs (after removing the subcortical ROIs and re-computing the Isomap), the distance to the origin of the Isomap space was computed and ranked from low (*left*) to high (*right*), with each region’s name shown on the *x* axis

Additional analyses were conducted for the connectome formed by 68 cortical ROIs alone (Fig. 7), with results confirming that in the absence of subcortical regions, regions including the precuneus, the paracentral lobule, the superior frontal gyrus, and the caudal anterior, posterior, and isthmus cingulate again are centrally mapped in the resulting Isomap embedding.

### Targeted node removal

To further understand the effects of node removal (beyond subcortical gray matter removal), we visually and quantitatively compared the resulting intrinsic geometry by removing rich-club nodes as defined previously [19], versus removal of randomly selected nodes of equal amount; for quantitative assessment we measured the degree of cohesion using the average distance to the center of the embedding ($\bar{d}$) to summarize the overall effect.

**Fig. 8:**
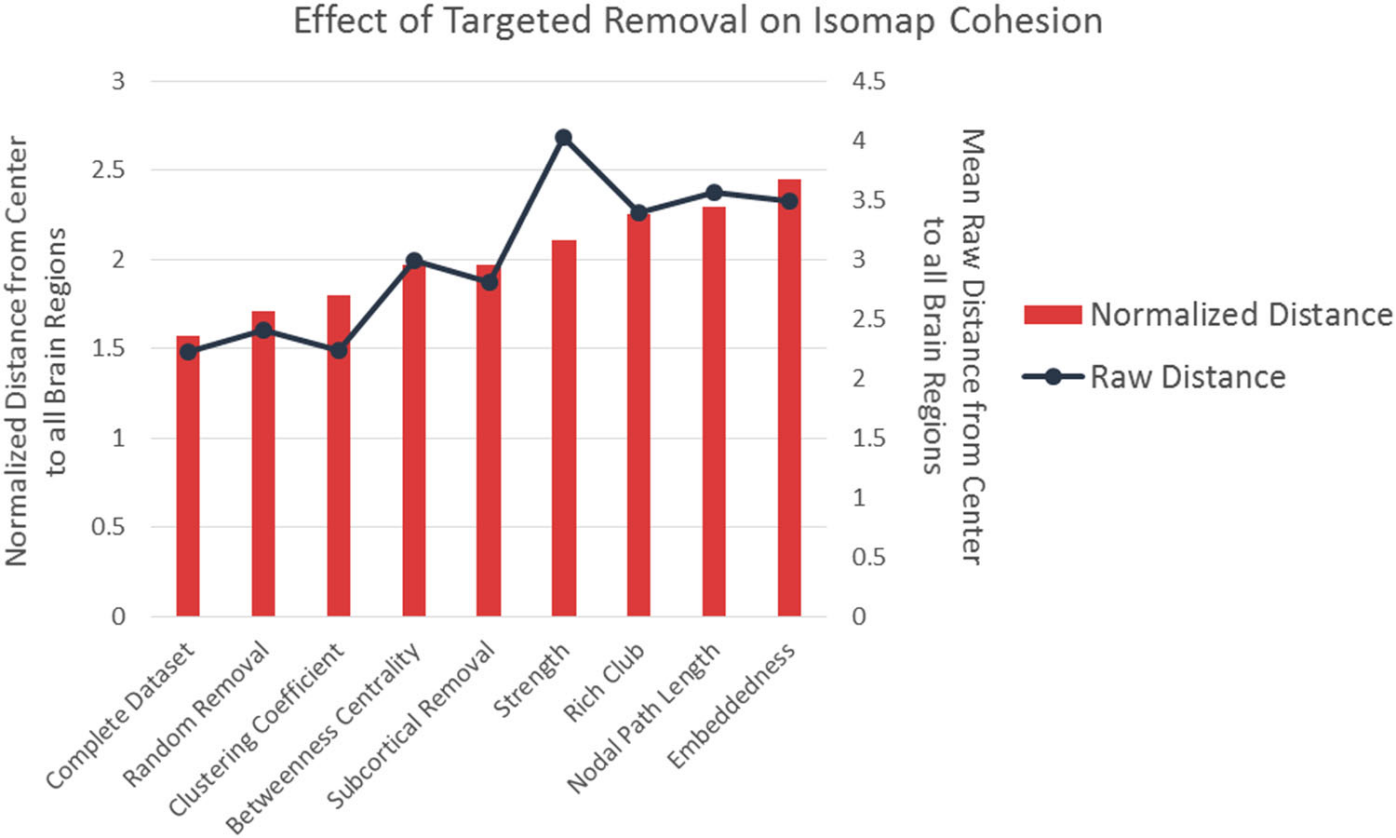
The mean (raw) distances and normalized distances (normalized by multiplying the average number of fibers between brain regions across the entire brain) measured from the center ($\bar{d}$) of the embedding to every point after various removal schemes. Random removal was repeated 20,000 times, and the average is shown. For raw distances, overall removing nodes that have the lowest clustering coefficient has the smallest impact, while removing nodes with the highest strength has the largest. However, comparing the normalized distances reveals that removing highly embedded nodes results in the largest normalized distance, while random removal has the smallest impact

Figure 8 shows the net results of 20,000 trials of random removal of 21.5 % of all nodes (the percentage of rich-club nodes after upsampling to the 620 node network). The mean and confidence interval for the Monte Carlo simulation was 2.411 and 0.249 (CI 5–95 %), respectively, while rich-club removal result is the third bar cluster from the right. As expected, rich-club removal has a larger impact on $\bar{d}$ as compared to random removal trials.

Similar simulations were then further conducted by removing an equal number of nodes (21.5 %) with respect to the following criteria: (a) nodal strength (high to low), (b) clustering (low to high), (c) nodal path length (low to high), (d) betweenness centrality (high to low), (e) subcortical region removal, and (f) embeddedness (high to low). Overall, removing nodes based on clustering minimally changes the cohesion of the connectome, supporting the fundamental differences between local properties such as clustering and global properties. By contrast, removing nodes with the highest degree of embeddedness has the largest impact on the cohesion of the connectome, thus supporting that highly embedded regions play important roles in the structural connectome.

**Fig. 9:**
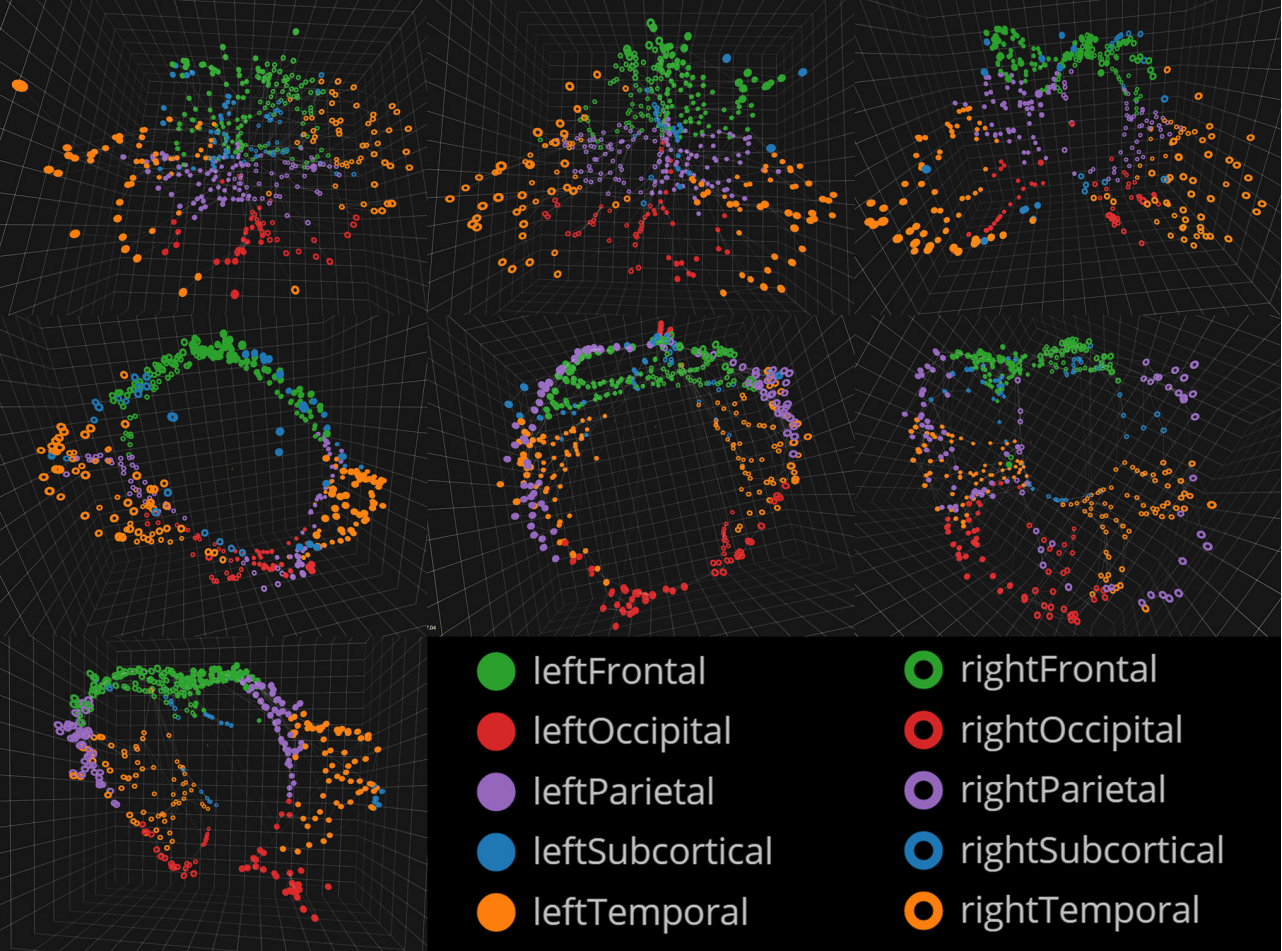
*Top row: Left* front view of an example Isomap embedding after 21.5 % of nodes were randomly removed. *Middle* Isomap embedding after removing nodes with the lowest 21.5 % clustering coefficient, *right* nodes removed according to the highest 21.5 % betweenness centrality. *Middle row: Left* removal of nodes with the lowest nodal path length. *Middle* removal of nodes according to the literature’s definition of rich club (21.5 % of all nodes; rich club regions include the* left* and* right* precuneus, superior frontal cortex, superior parietal cortex, hippocampus, putamen and thalamus). *Right* removal of 21.5 % of nodes with the greatest strength. *Bottom row: Left* removal of nodes with the top 21.5 % of embeddedness

Although plotting $\bar{d}$ under different removal strategies provides a quantitatively informative picture, visual investigations of these embeddings (Fig. 9) arguably provides an even more intuitive understanding of the impact of node removal. For example, the topology after rich-club removal (2nd row middle) or removal of nodes with the lowest 21.5 % nodal path length is ring-like as there is a loss in its central architecture. On the other hand, removing the top 21.5 % embedded regions (2nd row right) further degraded the structural connectome’s topology from a ring into a “horseshoe.” By contrast, a representative random removal showed minimal change in the shape of the embedding. These changes are interesting in the scope of brain exploration, but more importantly, could lead to a greater distinction of connectivity abnormalities in clinical cohorts or longitudinal changes in individual brains.

## Discussion

Using dimensionality-reduction techniques, we described a novel mathematical framework that creates 3D-embedded mappings representing the intrinsic geometry or topology of the human brain connectome. These embeddings can thus be thought of as the “native space” of brain connectome. Comparing results generated from tractography-derived structural connectome (Fig. 3), we showed that this intrinsic geometry only minimally relates to neuroanatomy. Thus, conventional visualization techniques that depict connectivity data in the neuroanatomic space may not be optimal (along similar lines, other recently proposed visualization techniques also used somewhat heuristic and arbitrary methods [2]).

Using two unique dimensionality-reduction algorithms, one linear (MDS) and one nonlinear (Isomap), we demonstrated the nonlinearity of this native space. Indeed, as shown in Fig. 4 the MDS-based 3D embedding exhibits a nonlinear relationship with the graph distance computed from the corresponding structural connectivity matrix, while the Isomap-based 3D embedding exhibits mostly a linear one. Moreover, visually MDS-based embedding suffers from the “crowding” problem (right panel of Fig. 3A), a known issue for linear techniques when the underlying geometry is nonlinear. This problem can be best understood using the famous “Swiss roll” example [5]. Here, the underlying intrinsic geometry of the Swiss roll is a 2D “sheet,” which is then rolled up and embedded in a higher-dimensional space (3D). As a result, the shortest 3D Euclidean distance between two points on the Swiss roll can be mistakenly much shorter than the true geodesic distance (i.e., the actual shortest distance needed to travel from one to the other if we reside in the space as defined by its intrinsic geometry). This thus creates “crowding” when a linear technique such as MDS is used, but could additionally entirely misrepresent the underlying true topology.

**Fig. 10:**
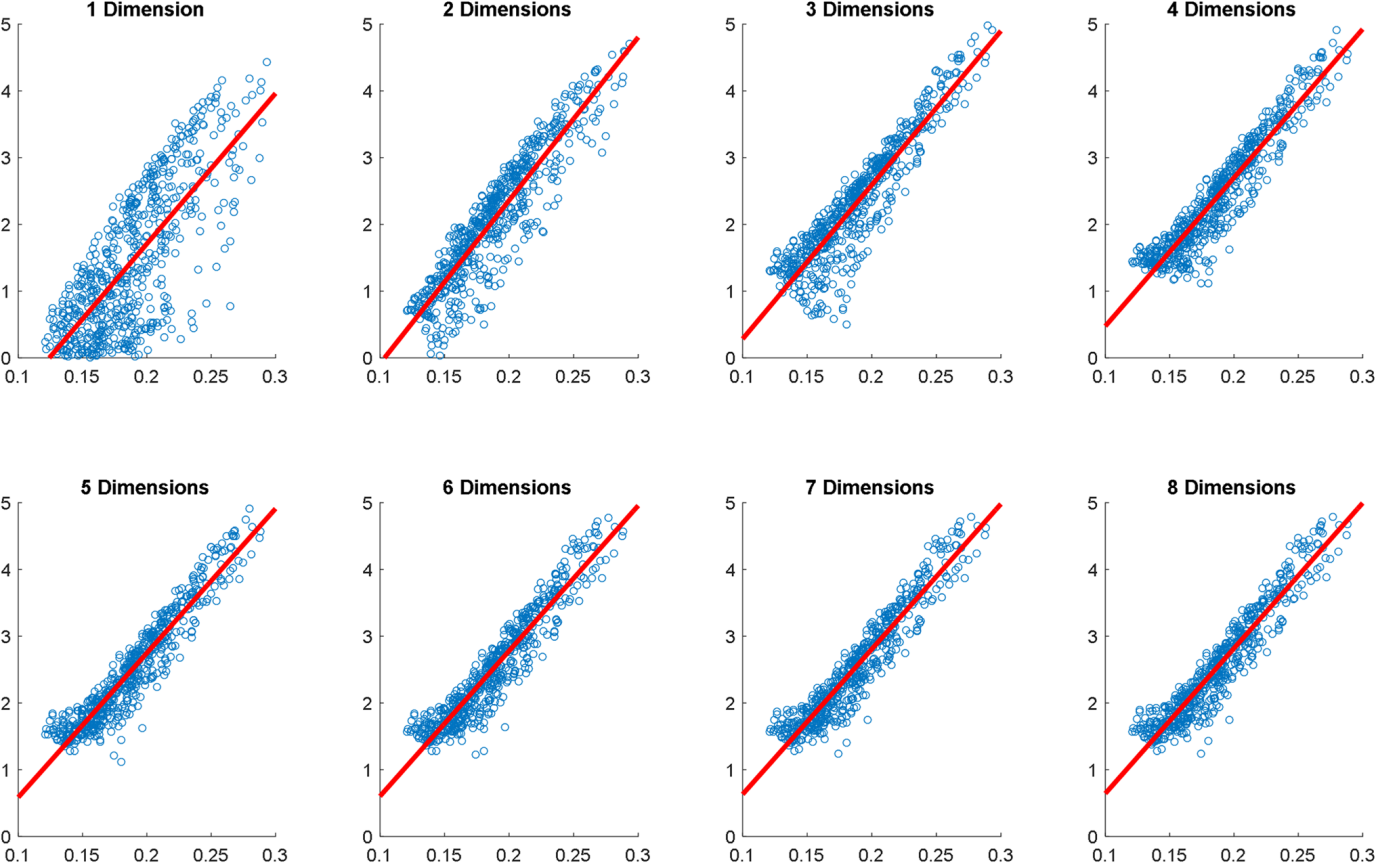
For the 620-ROI structural connectome, we computed the n-dimensional Isomap embedding (*n* ranges from 1 to 8) and plotted a region’s distance to the origin of the Isomap (*y*-axis) against its nodal path length 9 (*x*-axis). The root mean-squared errors (which can be interpreted as residual variances) for the dimensions from 1 to 8 are 0.708, 0.392, 0.359, 0.285, 0.260, 0.264, 0.254, and 0.253, respectively

At this point, one may ask the following interesting question. If we do not limit ourselves to 3-D embeddings due to their easier visualization, what is the optimal dimension in which the native space of the structural brain connectome could be best represented? To address this, we additionally conducted analyses similar to Fig. 5B, this time varying the number of dimensions of our Isomap embedding from 1 to 8 and computing the root mean squared error (RMSE) of the Euclidean distances to the origin of the embedding, after subtracting those accounted for by nodal path lengths. Results (Fig. 10) showed that the RMSE leveled off between *N* = 4 and 5, while *N* = 3 already accounted for a substantial amount of the residual variance (as measured using RMSE).

Next, using targeted attacks versus random node removal, we demonstrated the potential utility of the proposed framework, as this intrinsic geometry is sensitive to alterations in the underlying connectivity. Our approach may have clinical implications. For example, it can be used in surgical planning to quantitatively understand how lesions, either real or planned, can affect brain connectivity. Similarly, one may use such a visual representation to better detect differences in clinical cohorts, or to longitudinally track connectivity changes over time in individual brains.

The results of different removal strategies warrant further discussion. Note that removing nodes with the lowest 21.5 % of nodal clustering coefficient (right panel of the top row, Fig. 9) minimally impacts structural connectome’s intrinsic geometry, suggesting that clustering coefficient probes a network property that is to a great degree decoupled from properties such as nodal path length or strength. Also, the geometry of the brain connectome formed by the 68 cortical gray matter regions (i.e., removing Freesurfer-defined subcortical regions from the connectome) largely remains unaltered compared to the complete connectome formed by both cortical and subcortical regions. This relatively preserved efficiency of cortico-cortical communications (as evidenced by the intact shape of the geometry) thus suggests two parallel systems or “routes” of communication, one via the subcortical regions and the other entirely bypassing them. Based on these findings, we posit that one system (e.g., the subcortical routing) does not necessarily dominate the other; instead they work in conjunction and likely provide complementary functions to each other [26].

Another corollary to our findings is that regions that are mapped closer to the center in this native space are not all traditionally designated as having the rich-club property. Indeed, as shown in Figs. 6 and 7, the left and right caudate, pallidum, paracentral lobule, and caudal anterior, posterior, and isthmus cingulate are all relatively centrally located. Intuitively, one can hardly argue against the importance of these non rich-club nodes that consist of other subcortical regions (caudate and pallidum) [27–29], regions instrumental for sensori-motor function (paracentral lobule) [16, 30, 31], and regions known to be part of the limbic system (components of the cingulate) [32, 33]. Moreover, all these regions are considered highly embedded as recently shown in [23].

To provide an immersive visualization environment for these novel 3D representations, we have developed the BRAINtrinsic software, which is fully compatible with the Oculus Rift portable virtual reality technology (Oculus VR, Menlo Park, CA) (see Fig. 1 inset), thus allowing researchers to immerse themselves in these novel representations of brain connectivity through stereoscopic goggles. In order to help stimulate more research activities in this direction in the larger neuroimaging community, BRAINtrinsic is publicly available at http://creativecodinglab.github.io/BRAINtrinsic/.

## Conclusion

In this paper, we present a comprehensive treatment on the topology of the human brain connectome. While being novel, our framework outlined an entirely new way to conceptualize, visualize, and interact with connectivity data in its native space. In this space, the location and relative position of a region have intuitive interpretations in that the regions that are more important are more centrally embedded, while two regions that exhibit similar patterns of coupling with the rest of the brain are mapped near each other. The proposed framework could be easily adapted to multimodal data obtained from other types of brain imaging as well (e.g., EEG or MEG data). Examples of practical applications may also include subsequent changes in structure and function during normal development or monitoring disease progression in various neuropsychiatric disorders [34–38]. In addition, the topology of brain connectome after dimensionality reduction could be compared groupwise in disease states, and/or could be regressed with respect to various dimensional phenotypic measures.
